# Skin adaptation in lower limb amputees assessed through Raman spectroscopy and mechanical characterization

**DOI:** 10.1098/rsif.2024.0475

**Published:** 2025-01-08

**Authors:** Jack Hayes, Jennifer Andrews, Omar Abdelwahab, Tomas Andriuskevicius, Tom Briggs, Ralph Gordon, Peter Worsley, Claire A. Higgins, Marc Masen

**Affiliations:** ^1^ Department of Mechanical Engineering, Imperial College London, London, UK; ^2^ School of Health and Society, University of Salford, Manchester, UK; ^3^ Health Sciences, University of Southampton, Southampton, UK; ^4^ Department of Bioengineering, Imperial College London, London, UK

**Keywords:** confocal Raman spectroscopy, skin surface, stratum corneum, inflammation, indentation, friction

## Abstract

Following lower limb amputation residuum skin from the lower leg is used to reconstruct the residual limb. Unlike skin on the sole of the foot (plantar skin), leg skin is not inherently load bearing. Despite this, leg skin is required to be load bearing in the prosthetic socket. Current hypotheses propose that lower limb amputee skin can adapt to become load bearing with repeated prosthesis use. Here, we show using confocal Raman spectroscopy, mechanical characterization and cytokine analysis that adaptations occur which actually result in impaired barrier function, higher baseline inflammation, increased coefficient of friction and reduced stiffness. Our results demonstrate that repeated frictional trauma does not confer beneficial adaptations in amputee skin. We hypothesize that non-plantar skin lacks the biological capabilities to respond positively to repeated mechanical trauma in the same manner observed in plantar skin. This finding highlights the need for improved therapies as opposed to current mechanical conditioning or product solutions that directly relate to improving load-bearing capacity on the skin of lower limb amputees. This study also highlights the importance of measuring multiple parameters of application-specific skin at different scales for skin tribology applications.

## 1. Introduction

Skin plays an important role in how we perceive and interface with our environment [1] which can range from heat and moisture management to sensory or pain detection. When skin interacts with surfaces or medical devices for long periods of time the skin can break down, leading to the formation of blisters, ulcers and other lesions [2]. Skin is highly site-specific in its morphology and composition, driven by the specific functional requirements of skin on different body sites [3]. Foot skin is optimized for loading; pressures recorded during shod running can exceed 1000 kPa [4], yet plantar skin rarely breaks down. Skin on the surface of the fingertip has structures that allow the perception of roughness, adhesion and temperature of a surface [5]. The diverse range of roles required from different skin sites are reflected in equally diverse morphology [6] and mechanical performance [7].

The outermost layer of the epidermis, the stratum corneum, varies in thickness from 12 µm on the volar forearm [8] to over 1 mm in plantar skin on the sole of the foot [9]. The increased stratum corneum thickness and tougher underlying dermis of plantar skin enable the foot’s role in load bearing and locomotion [10]. Computational knockout models of plantar and non-plantar skin in static compression and shear have shown that the morphology and composition of plantar skin provide additional protection by reducing internal strains [10]. In particular, the plantar geometry of a thick stratum corneum helps reduce pressure in the underlying dermis [10]. The plantar composition of both the epidermis and dermis resists distortion and helps prevent deformation of the tissue [10]. Positive adaptations are also seen in the plantar epidermis, which thickens and stiffens in response to repeated mechanical loading [11].

One group that suffers with persistent skin breakdown is lower limb amputees. Skin injuries in amputees are very prevalent, with 63% of amputees reporting one or more skin injuries in the month prior to survey response [12]. Skin injuries in lower limb amputees is a significant problem that is set to increase with increasing rates of vascular diseases. Globally, there is an amputation every 30 s due to a non-healing diabetic ulcer [13]. A lower limb amputee’s residuum utilizes leg skin from the wound site to create the residuum that will interact with the prosthetic socket. The lateral residuum skin of the amputee interfaces with a prosthetic socket through shear loading to support the weight of the amputee, keep the device in place and allow the user to walk. During standing, socket interface pressures have been found to be 149 kPa and longitudinal shear 56 kPa, respectively, while during heel strike socket interface pressures were recorded in excess of 300 kPa and longitudinal shear in excess of 100 kPa [14,15]. This indicates there is a substantial contribution of shear stress at the interface of a prosthetic socket which creates a unique scenario, where non-plantar skin is now required to function in a load-bearing capacity.

Shear at the prosthetic socket–skin interface is an important factor in maintaining adequate fit and reducing slip between the device and skin [16]. However, shear forces are known to contribute significantly to soft tissue injury [17]. Previous work has found that non-plantar leg skin of healthy participants adapts to frictional trauma with observations of decreasing coefficient of friction, decreased skin temperature, reduced sensation [18] and formation of thicker collagen fibres [19]. Comparatively, corneal epithelial cells subjected to frictional trauma *in vitro* showed higher levels of inflammation [20] and apoptosis [21] than only normal loading. There is therefore confounding information on whether adaptation occurs in skin at non-plantar sites. Further still, despite skin adaptation hypotheses, skin injuries remain high even years after amputation [22].

Residuum skin, unlike plantar skin, does not have specialist structures or composition to resist distortion and deformation [10]. Short-term non-plantar skin conditioning (two weeks) in an able-bodied population led to no statistical differences in blood vessel density in the dermis and minimal increases in epidermal thickness [23]. In transtibial amputees, increases in limb stiffness have been recorded at the patellar tendon while decreases in limb stiffness are found in posterior and lateral positions. Residual limbs also have higher adipose composition compared with contralateral limbs [24]. If biological machinery allowing skin adaptation to loading exists within non-plantar skin, in the same manner observed in plantar skin, recordings of increased stratum corneum thickness and increased stiffness in indentation should be observed and contribute positively to load bearing. The current hypothesis is that lower limb residuum skin can adapt to become load bearing with mechanical conditioning [19]. However, lower limb amputees suffer a disproportionately higher skin injury rate (65%) when compared with the general population [25]. It has been shown that in transtibial amputees there is muscle atrophy, increased adipose infiltration and subsequent softening of residual limbs with respect to intact contralateral limbs [24], but morphology was only recorded on the sub-dermal scale and not the epidermal scale.

Research on parameters relating to mechanics and morphology of residuum skin are lacking [26]. Research into skin barrier function in lower limb amputees has indicated reductions in transepidermal water loss [27], and it has been consistently reported that dermatoses are prevalent in lower limb amputees. The mechanical interactions of the prosthetic socket with the epidermis are yet to be explored.

In this body of work, we sought to better understand the mechanics, morphology and molecular composition of lower limb amputee skin and assess what adaptations had occurred from the unloaded intact skin, loaded amputee skin and loaded skin on the plantar heel. *In vivo* analysis of skin composition, morphology and mechanical properties were performed on the intact limb skin, the residual limb skin and plantar heel of 13 lower limb amputees. The analysis of the results is compared to determine if statistical differences exist between the unloaded contralateral limb and loaded residuum skin, and see if any of these differences suggest adaptation to a load-bearing (plantar skin) phenotype.

These results help bridge understanding on if the skin on the residual limb can adapt to a new role of load-bearing skin. Previous research has highlighted the importance of morphology and composition in *in silico* analysis, while the present study aims to explore morphology and mechanics experimentally.

## 2. Material and methods

*In vivo* analysis of skin composition, morphology and mechanical properties were performed on the intact limb skin, residual limb skin and plantar heel skin of seven transfemoral and six transtibial lower limb amputees, all using suspension socket prosthesis. Case-controlled analysis was conducted to identify differences between non-loading sites (intact limb) and an adapted loaded site (residual limb) against a loading site (plantar heel). The present study aims to explore skin morphology and mechanics experimentally. Moreover, the barrier function and state of inflammation are also investigated.

The study protocol was reviewed and approved by the University of Salford ethics panel (application 6751) and a data sharing agreement was established between the University of Salford, University of Southampton and Imperial College London. Participants were recruited through the University of Salford’s professional patients; measurements were taken following their written informed consent to take part in the study. Participants were recruited if they had lower limb amputation and had no current wounds on any skin sites used for measurements. In total, 13 participants were recruited. All participants were male and had an average age of 64.92 ± 21 years. A summary of participant demographics is provided in table 1.

**Table 1.** Participant demographics.

participant ID	age	amputation site	cause of amputation	years since amputation	other pathologies
TF01	74	right leg above knee	trauma	57	none
TF02	69	right leg above knee	vascular (blood clot)	5	none
TF03	43	right through knee	congenital	43	none
TF04	60	right leg above knee	vascular+type 1 diabetes	2	diabetes (type 1)
TF05	80	right leg above knee	cancer	52	none
TF06	65	left leg above knee	trauma	47	none
TF07	52	left leg above knee	vascular	1	diabetes (type 1)
TT01	58	right leg below knee	did not comment	20	did not comment
TT02	61	right leg below knee	trauma	42	none
TT03	85	left leg below knee	trauma	25	none
TT04	69	left leg below knee	vascular (blood clot)	7	diabetes (type 2)
TT05	63	left leg below knee	trauma	20	diabetes (type 2)
TT06	58	right leg below knee	trauma	25	none

**Figure 1.**
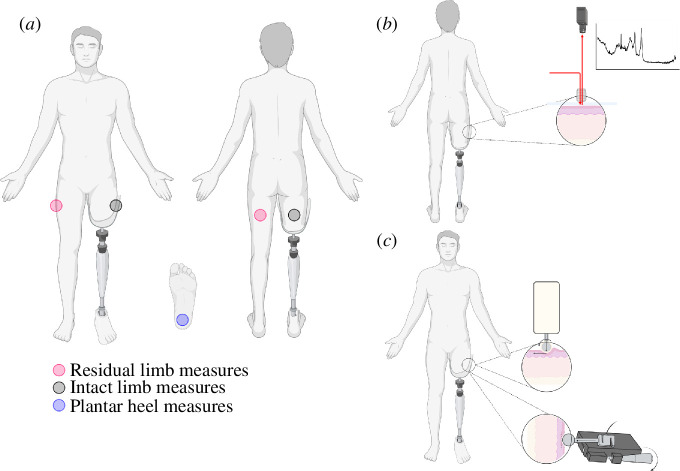
Study outline. (*a*) Measurement locations for the study. (*b*) Raman spectroscopy measurement locations and principle. (*c*) Tribometer and indentation measurement sites.

Participants underwent a seated 15 min acclimatization period to ensure transient effects of adaptation to the environment were not recorded. Following the acclimatization period, participants’ measurements (residual limb first, intact limb second and plantar heel third) were conducted as least invasive first to most invasive last, as hydration, molecular composition and morphology are sensitive to mechanical stimuli [17,28–30]. We therefore record these measurements first and performed sebutape analysis, mechanical indentation and friction quantification after to avoid affecting the previous measurements. We use non-invasive imaging techniques to measure hydration, molecular composition and morphology. Measurements were taken from all 13 participants at the lateral loaded surface on the residual limb and intact limb, and eight patients for the plantar heel. Figure 1 illustrates the various measurements and the locations at which these measurements were performed.

### 2.1. Morphological measures

Skin surface roughness measurements (6 × 8 mm) were taken with a UVA-light camera (Visioscan VC 98, Courage+Khazaka electronic GmbH, Germany) [31]. Evaluation of the image is conducted using a built-in software SELS (Surface Evaluation of the Living Skin). This method uses image processing to define a number of parameters relevant to skin. As a measure of apparent skin roughness the SEr parameter is used, which is calculated from the image contrast in peaks and troughs of skin. The lower the SEr value, the lower the apparent roughness.

Stratum corneum thickness was determined using the RiverDiagnostics Gen2-SCA confocal Raman system. The 785 nm laser is capable of measuring Raman spectra of wavelengths associated with water, while spectra are analysed using built-in software SkinTools. Previous work has shown that the set-up can be used to determine stratum corneum thickness [32]. Briefly, stratum corneum thickness is defined as the depth point at which the first derivative of the hydration profile is zero, implying water content is at a local stationary maximum [32], and the method for determining the skin surface has been explored in the literature [33].

### 2.2. Mechanical measures

A custom-made measurement device was used to record *in situ* force–displacement curves. A rigid 10 mm radius stainless steel indenter is contacted with the skin and displacement was increased by turning a microscopic lead screw until reaching 5 mm of indentation, while the force is measured using a force transducer (Applied Measurements, DBBSMM load cell with signal conditioner SGA), with data signal recorded and stored using a DAQ system (National Instruments, NI USB-6001). Each measurement was performed five times on the lateral surface of the residual limb, the equivalent site on the intact limb and the plantar aspect of the heel, as indicated in figure 1. Indentation stiffness was determined assuming a linear relationship between force and displacement (*F* = *K*⋅*d*), where *F* is the force of indentation, *K* is the stiffness of the substrate and *d* is the displacement.

A mobile tribometer suitable to measure skin friction with a controlled and variable normal load (range 0.5−2.0 N) and velocity (range 1−10 mm s^−1^) was utilized for friction measurements. An overview of instrumentation and data analysis has been established in [34]. Briefly, the device initiates one clockwise rotation, recording friction force and applied normal force. Dynamic coefficient of friction is then calculated with the measured friction force and normal force when the wheel is in motion at velocity *ω*. The specimen in sliding contact with the skin is covered with prosthetic liner material, representing the friction condition at the residuum interface. Friction data were computed by analysing the middle 50% of the motion to only account for when the contacting specimen was in steady-state sliding motion against the skin.

### 2.3. Molecular measures

*In vivo* Raman spectra were obtained with the RiverDiagnostics gen2-SCA 785 nm laser source (for measurements in the 400–2500 cm^–1^ spectral region) and 671 nm laser source (for measurements in the 2500–4000 cm^–1^ spectral region). The measurement stage is an inverted microscope stage, which collects Raman spectra from emitted light to generate depth profile spectra. The confocal Raman set-up contains an immersion microscope objective which focuses laser light on the skin through an optical window on which the skin rests. The objective also collects Raman scattered light from the skin. Data are recorded at depths of 0–33 µm for all skin sites, skin is contacted with the window stage and 5 µm diameter areas of skin are analysed. One spectrum is recorded at each depth step size (table 2) with five repeat measures taken for each participant to get representative Raman spectra from each skin site.

**Table 2.** Confocal Raman spectroscopy experimental procedure.

locations	track (µm)	step size (µm)	exposure time (s)
residual limb, intact limb and plantar heel	0–33	3	5

Sebum components such as inflammatory cytokines have been analysed using sebutape in a variety of skin tissue studies. The present study utilizes a previously developed method to analyse sebum contents [29]. Briefly, Sebutapes (Cu-Derm, Dallas, TX, USA) were applied to skin for 2 min collection, using gloved hands to avoid cross-contamination of skin proteins. The adhesive side of the tape was placed directly on the skin site. In all cases, after the 2 min collection interval, the Sebutape was removed using blunt forceps and stored in Eppendorf vials at −80°C until analysis. On the analysis day, 850 µl of 0.1% DDM (*n*-dodecyl-d-maltoside, Cat. no. 89902, ThermoFisher) was added to each Sebutape followed by 1 hour shaking and brief sonication and centrifugation to extract the cytokine samples. Cytokine levels were measured using chemiluminescent multi-analyte V-PLEX kit (Cat. no. N05049A-1, MSD) according to the manufacturer instructions. Pro-inflammatory cytokines were profiled, affiliated with skin inflammation caused by mechanical insults which included IFN-γ, IL-10, IL-12p70, IL-13, IL-1β, IL-2, IL-4, IL-6, IL-8 and TNF-α [35].

All Raman spectroscopy results presented were filtered by removing data outside ±1.5 interquartile range at each depth point, this analysis step eliminates the effect of cosmic ray artefacts. All Raman data are then tested for normality using the Shapiro–Wilk test. Datasets with high significance (*p* < 0.05) are determined not to be normally distributed, and non-parametric paired tests for statistical significance must be used. All spectra for all skin sites were found to be not normally distributed (*p* < 0.05). All Raman results stated as significant have significance values of 5% (*p* < 0.05), as determined by a Wilcoxon test.

Results relating to stratum corneum thickness, apparent skin roughness, coefficient of friction, stiffness and cytokine analysis were assumed to be normally distributed and were tested using a two-tailed paired *t*‐test, all results stated as significant have significance values of 5% (*p* < 0.05).

## 3. Results

### 3.1. Morphological measures

**Figure 2.**
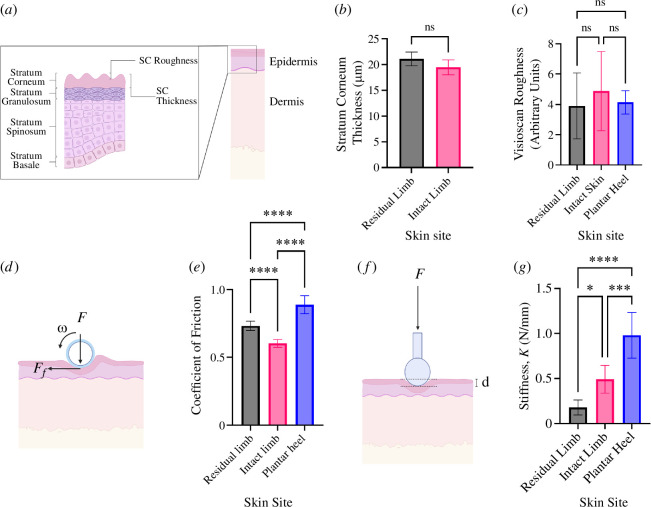
Morphological and mechanical characterization of skin. (*a*) Skin layer schematic. (*b*) Stratum corneum thickness. (*c*) Apparent skin surface roughness. (*d*) Schematic of mobile tribometer measurements. (*e*) Dynamic coefficient of friction measurements. (*f*) Schematic of limb stiffness measurements. (*g*) Stiffness measures from 5 mm of indentation. The schematics in (*a*), (*d*) and (*f*) were created using BioRender. In all cases residual limb is plotted as grey, intact limb in pink and plantar heel as blue, whiskers represent confidence intervals (5–95%). ****p < 0.0001, ***p < 0.001, *p < 0.05 and ns is *p* > 0.05.

Previously, confocal Raman spectroscopy has been utilized to non-invasively detect the spatial distribution of water [36], epidermal components [37] and stratum corneum thickness [32]. This study aims to find multi-scale differences in skin sites with a relation to mechanical parameters and evaluate the degree of adaptation by residuum skin to loading. It has been hypothesized that, in response to shear and contact pressure, collagen fibres would adapt to loading by increasing in diameter and confer better load-bearing capacity [19]. Here, confocal Raman spectroscopy was used to measure stratum corneum thickness, to understand to what degree amputee skin’s morphology is altered by repeated mechanical loading. The average stratum corneum thickness of the intact limb and residual limb were found to be 19.5 µm (s.d. 5.05) and 21.1 µm (s.d. 4.96), respectively (figure 2*b*). In the plantar heel, the stratum corneum is greater than 900 µm in histological images [10] and hence measurements to a depth of 33 µm were still in the stratum corneum of the plantar heel measures and could not tell us about stratum corneum thickness. The comparison of the residual limb and intact limb highlights a small increase of stratum corneum thickness of 1.6 µm, however, this difference is not statistically significant (*p* > 0.05) (figure 2*b*).

Roughness measures taken using a Visioscan VC 98 revealed that the residuum is smoother than the intact limb and plantar heel at 3.9 arb. units (s.d. 3.6) compared with 4.9 arb. units (s.d. 4.3) and 4.1 (s.d. 1.2), respectively, but this difference is also not significant (*p* > 0.05) (figure 2*c*).

### 3.2. Mechanical measures

Frictional interactions are influenced by adhesion and deformation of the skin. Details about how different skin sites perform with respect to coefficient of friction is an active area of research; however, there is currently a lack of research with a focus on amputee skin. Using a previously developed tribometer [34] it was found that the coefficient of friction is significantly higher (*p* < 0.0001) at the residual limb at 0.7 (s.d. 0.76) compared with 0.6 (s.d. 0.58) on the contralateral limb. Both of these measurements are significantly (*p* < 0.0001) lower than the recorded friction on the plantar heel at 0.9 (s.d. 1.06) (figure 2*e*).

Mechanics of the skin play a critical role in protecting the tissue during locomotion. Stratum corneum mechanics protect from cracking [38], and epidermal and dermal mechanics help protect skin from forming pressure ulcers [10]. Using a custom-made indenter to measure tissue mechanics it was found that the stiffness of the residual limb was 0.18 N mm^−1^ (s.d. 0.13), which was significantly lower than the intact limb (*p* < 0.001) and plantar heel with relative stiffnesses of 0.49 N mm^−1^ (s.d. 0.24) and 0.98 N mm^−1^ (s.d. 0.38) respectively (figure 2*g*).

### 3.3. Confocal Raman spectroscopy measures

**Figure 3.**
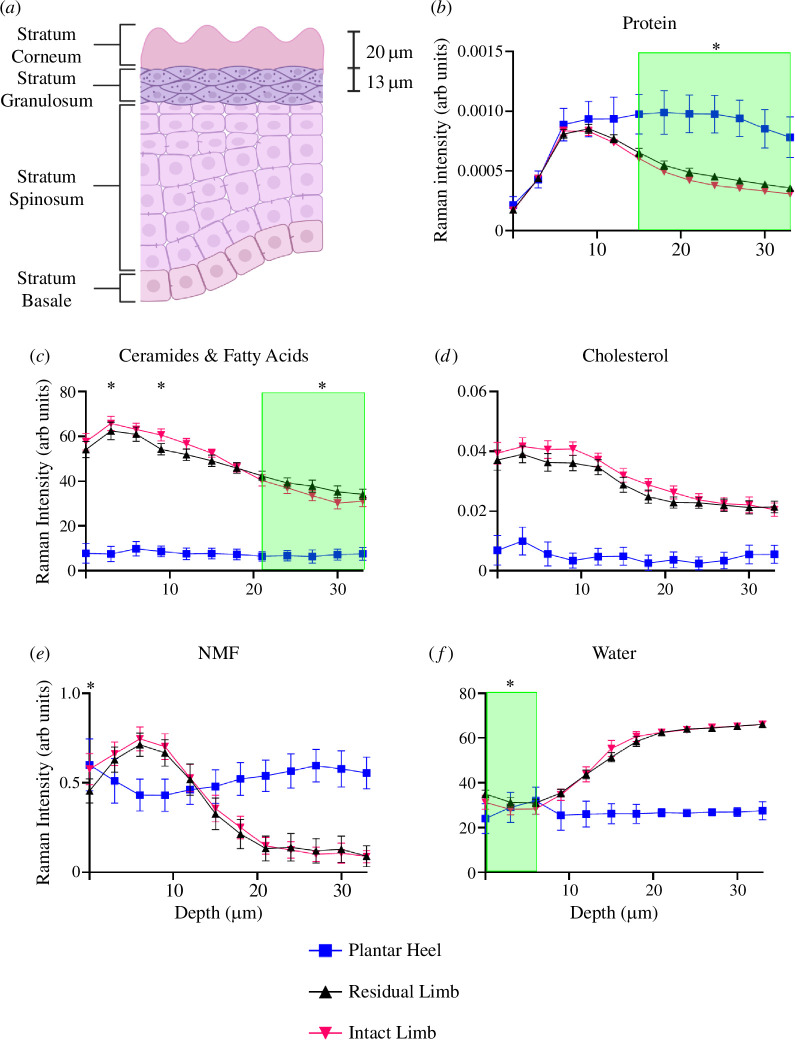
Raman spectra profiles across the depth of skin. (*a*) Skin layer schematic with depth profiles next to approximate skin layers. (*b*) Proteins. (*c*) Ceramides and fatty acids. (*d*) Cholesterol. (*e*) Natural moisturizing factor (NMF). (*f*) Water. The diagram in (*a*) was created using BioRender. In all cases, residual limb is plotted as black, intact limb in pink and plantar heel as blue. In all cases, each point represents the median value and whiskers represent confidence intervals (5–95%). Green boxes indicate continuous regions of statistical significance, *p* < 0.05. * Indicate single depths of statistical significance, *p* < 0.05, when comparing the intact limb with the residual limb.

The data so far suggests that surface properties of amputee skin remain unaltered by prosthetic use and that the interface mechanics of the limb show higher friction and reduced limb stiffness. These findings would suggest that greater deformation and distortion are exerted on amputee skin during prosthesis use, which might impact its barrier function. To better understand the barrier function in lower limb amputees, Raman spectra were taken from the residual limb and the intact limb, in addition to plantar heel spectra, to quantify residual limb skin transition to a plantar identity. Measurements were taken from 0–33 µm depths, which encompass the stratum corneum and granulosum layers on the residual limb and intact limb, but only the superficial stratum corneum on plantar skin. It was found that the residual limb has higher protein content at depths 15–33 µm from the skin’s surface, when compared with the same depth in the intact limb (*p* < 0.05) but exhibits half the protein levels in the same depth (15–33 µm) as the plantar heel (figure 3*b*). Total lipid contents (ceramide and fatty acids) were significantly (*p* < 0.05) lower in the stratum corneum (3 and 9 µm depth) and granular layers (21–33 µm depth) of the epidermis, in residual limb skin compared with the intact limb (figure 3*c*). The plantar heel exhibited a sevenfold reduction in lipid content (ceramide, fatty acid and cholesterol) across the first 33 µm compared with the residual limb. The stratum corneum at the residual limb had lower cholesterol content than intact limb skin across the first 33 µm of the epidermis but these differences were not significant (*p* > 0.05) (figure 3*d*). Natural moisturizing factor (NMF) was reduced at the residuum skin surface compared with the intact limb (*p* < 0.05) (figure 3*e*). In both the intact limb and residual limb, NMF increased in the upper stratum corneum before decreasing in the stratum granulosum. The plantar heel exhibited comparable NMF content at the skin surface as the residual and intact limbs, which remained high throughout all the measured depths. It was found that the water profile of the residual limb was significantly higher than the intact limb at the skin surface and just below (0–6 µm depth) (*p* < 0.05) (figure 3*f*). In comparison, the water profile of the plantar heel is similar at the skin surface and just below (0–6 µm depth) but remains flat across 33 µm.

### 3.4. Cytokine measures

Inflammation is the skin’s earliest response to mechanical damage, and, with the presented results on reductions in key epidermal barrier components (figure 3*b*–f), baseline inflammation is likely to be elevated at the residuum. In stage 1 pressure ulcers on the sacrum, IL-1β, IL-6, IL-8, TNF-α and IFN-γ were found to be upregulated [35]. To better understand if uninjured lower limb amputee skin is inflamed, Sebutapes were applied to the skin at the residual limb and intact limb to collect sebum components for cytokine analysis [29].

**Figure 4.**
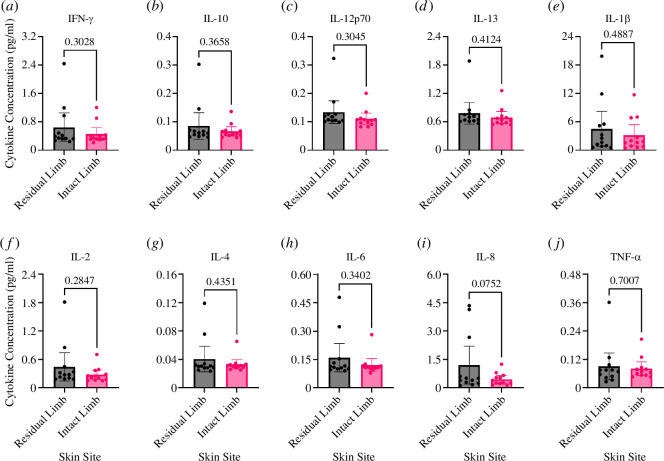
Cytokine expression of entire study sample. (*a*) IFN-γ. (*b*) IL-10. (*c*) IL-12p70. (*d*) IL-13. (*e*) IL-1β. (*f*) IL−2. (*g*) IL-4. (*h*) IL-6. (*i*) IL-8. (*j*) TNF-α.

It was found that after acclimatization the skin on the residual limb had higher levels of baseline inflammatory cytokines in their sebum (IFN-γ, IL-10, IL-12p70, IL-13, IL-1β, IL-2, IL-4, IL-6, IL-8 and TNF-α) compared with sebum from the skin on the contralateral limb. However, these differences were not statistically significant (*p* > 0.05) (figure 4).

When analysing the largest fold-changes for each cytokine, the three largest upregulations were IL-8 at 0.4539 pg ml^−1^ versus 1.198 pg ml^−1^, IL-2 was the second most at 0.2759 pg ml^−1^ versus 0.4424 pg ml^−1^ and IL-1β 3.165 pg ml^−1^ versus 4.509 pg ml^−1^ for the intact limb and residual limb, respectively.

## 4. Discussion

In this study, we set out to test the hypothesis that skin adaptation occurs in residuum skin as a result of prosthetic use. Although this is a long-held belief, it is at odds with high injury statistics for lower limb amputees that are reported in the literature [12,22,25]. Understanding of amputee lower limb mechanics are lacking, and tribology of the residual lower limb has only been minimally investigated. Epidermal architecture of lower limb amputees had not been explored. We therefore set out to characterize skin on the residual limb versus the contralateral intact limb, using morphological, mechanical and molecular measurements and compared this with the plantar heel to investigate any transitions to a load-bearing plantar phenotype.

### 4.1. Stratum corneum thickness and roughness

Using confocal Raman spectroscopy to measure stratum corneum depth, and a Visioscan VC 98 to measure roughness, we found a small (1.6 µm) increase in thickness and decrease in roughness on the residuum when compared with the intact limb; however, these changes were not significant (figure 2*b*,*c*). While an increase in residuum skin was recorded in our study, epidermal thickness on the plantar heel has been measured using ultrasound at 660 µm *in vivo* [39] and approximately 900 µm in *ex vivo* histology [10], suggesting the transition to a load-bearing identity is limited with respect to stratum corneum thickness. This counters literature suggesting that the stratum corneum can significantly thicken in response to loading [11]. This prior study demonstrating human skin adaptation was evaluating plantar skin on the sole of the foot [11], while in non-plantar studies in pig and rabbit models, there were observations of increased collagen fibre diameter, increased epidermal thickness and decreased collagen fibre density [40,41].

To our knowledge, no studies have demonstrated beneficial adaptations in human non-plantar skin. In human lateral tibia skin, frictional trauma has been shown to lead to increases in skin roughness [18]. However, the timescale of that study was limited to a month in comparison with the long-term exposure to mechanical insult that would have occurred in our study participants (mean time since amputation = 27 years). Skin roughness varies substantially between plantar and non-plantar skin sites; roughness of finger pads have been reported at 26.1 µm whereas non-plantar skin such as the ventral forearm has roughness values of 12 µm [42]. Our results suggest that residuum skin is comparable in visioscan roughness to the plantar heel.

### 4.2. Epidermal architecture

Although the confocal Raman spectroscopy revealed no significant differences in stratum corneum thickness, it did show several differences in stratum corneum and granular layer composition between amputee residuum skin and intact limb skin. All measured metrics, which included protein, lipid, NMF and water content, were significantly different in the amputee residuum skin versus intact limb skin, at specific depths beneath the skin surface.

First, our results show increased protein levels in the deeper stratum corneum and granular layer of amputee skin compared with the contralateral limb (figure 3*b*). Keratin proteins form 80% of the stratum corneum’s dry weight [33] and provide mechanical integrity to corneocytes [43]. In corneocytes, keratin, in the presence of NMF, binds water into corneocytes [43], allowing effective desquamation (shedding of surface corneocytes) of terminal keratinocytes [44]. Our results also show that lower limb amputee skin has lower levels of NMF in the superficial levels of the stratum corneum, compared with skin on the intact limb. Previous studies have shown that the combination of mechanical trauma and occlusion leads to significant reductions in free amino acids (a major constituent of NMF) compared with just mechanical trauma or occlusion [45]. It has also been reported that 100% humidity and very low humidity atmospheres blocked the proteolysis of filaggrin and subsequent formation of NMF [46]. Other studies have found that frictional trauma can remove superficial NMF [47]. Since the prosthetic socket is a high-humidity, and high-friction environment, any or all of these mechanisms could be contributing to the reduced NMF seen in the amputee skin in our study.

A complex interplay of lipids, proteins and NMF are important in maintaining the barrier function of the stratum corneum [43]. In addition to proteins and NMF, our confocal Raman spectroscopy analysis showed that lower limb amputee skin has lower cholesterol, ceramides and fatty acids (major constituents of the lipid matrix in the stratum corneum) across the depth of the stratum corneum when compared with equivalent depths in skin on the intact limb. Lipids are known to play an important role in stratum corneum architecture, retention of water within the stratum corneum, controlled movement of water and roles in cellular processes like keratinocyte differentiation and inflammation [48,49]. Previous work has shown that decreases from baseline levels of ceramide and cholesterol are a symptom of atopic dermatitis, where there is an impaired barrier function [50]. If the lipid barrier is disrupted and water is distributed in the upper layers of the stratum corneum, water can cause corneocyte swelling and disruption of intercellular lipid bilayers. The confocal Raman spectroscopy results also show an increase in water content at the skin surface on the residuum. Deviations from the assumed healthy profile of the intact limb would have downstream effects on cellular function in the stratum corneum and reduce barrier function at the residuum–prosthetic interface. The presence of water also softens skin mechanics through degradation of desmosomes [45] leading to higher skin friction [51] and enhanced permeability leading to a greater chance of infection and irritation [52]. The reported findings would suggest impaired homeostasis and impaired response to infection, inflammation and wounding. During barrier disruption, it has been previously reported that ceramide, cholesterol and fatty acid levels increase through basal keratinocyte scavenger receptor class B-1 to restore the barrier [47,53]. Lipid content is reduced in superficial layers, therefore such mechanisms appear to be insufficient to maintain ceramide, cholesterol and fatty acid levels in longer-term frictional trauma (figure 3*c*,*d*). Our observations of reduced lipid content would disrupt water profiles in the stratum corneum and lead to poorer barrier function and impaired inflammatory response. Our measures of residual limb skin compared with the plantar heel highlight some adaptations towards a plantar phenotype. However, the changes in epidermal content are still substantially lower in lipid content and substantially higher in protein content when compared with the plantar heel.

### 4.3. Mechanics

The observed loss of superficial skin substances such as lipids and NMF, which impact lubrication, and an increase in superficial water content, might also explain the higher friction recorded in our study (figure 2*e*). Previous investigations into skin friction have reported coefficient of friction at around 0.5 [18,42,54] for non-plantar skin compared with 1.2 for the plantar heel [55]. While the precise friction measurement in any given study can be influenced by several factors such as intrapersonal and hydration differences, making it difficult to compare between datasets, our results showed that friction was significantly higher at the residual limb (0.7) compared with the intact limb (0.6), indicating a transition in frictional behaviour in residuum skin towards that of a plantar identity, which we record at 0.9 for the plantar heel. In the short term, frictional trauma has been shown to lead to increases in superficial cholesterol [47]. We find that in our study, where there has been longer-term frictional trauma, there were reductions in lipid content near the surface of the stratum corneum. It has previously been investigated that lipid content has minimal effects on friction, whereas reduced moisture content in the stratum corneum increases the stiffness of the skin, which causes a reduction in friction through the reduced contact area [56]. Our results agree with previous work that an increase in moisture at the superficial layers of the stratum corneum (figure 3*f*) can lead to increased skin friction (figure 2*e*), and in our study we see this manifested in the residual limb compared with the intact limb.

In addition to friction, we also conducted mechanical testing to evaluate tissue stiffness. In plantar skin, observations of higher mechanical properties are linked to reduced internal strain in the tissue, with previous reports finding that plantar skin deforms 1.6 times less under compression than non-plantar skin [10]. To support the loads transferred through the prosthesis to the body during movement requires resilience from the skin. It has previously been shown that the increased stiffness of plantar skin protects the tissue from superficial pressure injuries like pressure ulcers, by reducing internal compressive and shear strains [10]. Our results indicate that residuum skin actually becomes softer in indentation as a result of prosthesis use (figure 2*g*), suggesting greater susceptibility to injury from device use owing to higher internal strains. A reduction of residuum mechanical properties has previously been reported in deep tissue with greater infiltrating adipose tissue in transtibial amputees and softer mechanics observed in the residuum than intact limbs [24]. Our findings support these previous observations, and show that this reduction in mechanical properties is also coupled with increased friction, a finding our results support across our recruitment sample of both transtibial and transfemoral amputees. Overall, we find that the contact situation is deleterious to optimal skin health with a very soft contact and high friction recorded at the residual limb relative to the intact limb (figure 2*e*,*g*).

### 4.4. Inflammation

Finally, we evaluated the levels of the pro-inflammatory cytokines, finding higher, but not significant, levels in the sebum from amputee residuum skin versus the intact limb. These cytokines are usually upregulated in wound environments in association with inflammatory status. While, in the current study, there were no skin wounds on participants at the time of the study, skin injury on the residuum is a common problem for amputees. Our results suggest that the amputee residuum skin is in a state of baseline inflammation, perhaps caused by repeated injury, or mechanical loading of the site when compared with the skin on the intact limb [35]. A similar phenotype has recently been reported in plantar weight-bearing skin, meaning plantar skin resembles a wounded phenotype and as such shows impaired wound healing [57]. In addition, such a higher baseline inflammatory profile reflects a higher risk of chronic non-healing skin injury at the residuum–prosthetic interface [58], highlighting the risks posed to individuals with lower limb difference.

Overall, several new insights were gained into the *in vivo* state of lower limb amputee skin, and this study may form the basis for larger *in vivo* studies to be conducted on the lower limb difference population. There are, however, some limitations to the study. The participants in the present study form a small sample size and exhibit some heterogeneity in age, amputation level and time since amputation. Our study focuses on how prosthesis use affects skin surface properties and, in particular, stratum corneum and epidermal adaptations with regard to molecular composition, morphology and mechanical response to load. Greater understanding of dermal and deeper tissue adaptations would be beneficial to the field. Previous work has investigated bulk adaptations to amputee residual limbs using magnetic resonance imaging (MRI), while the work presented here focuses on very superficial adaptations of the residuum. Bridging these two scales warrants further investigation.

## 5. Conclusion

**Figure 5.**
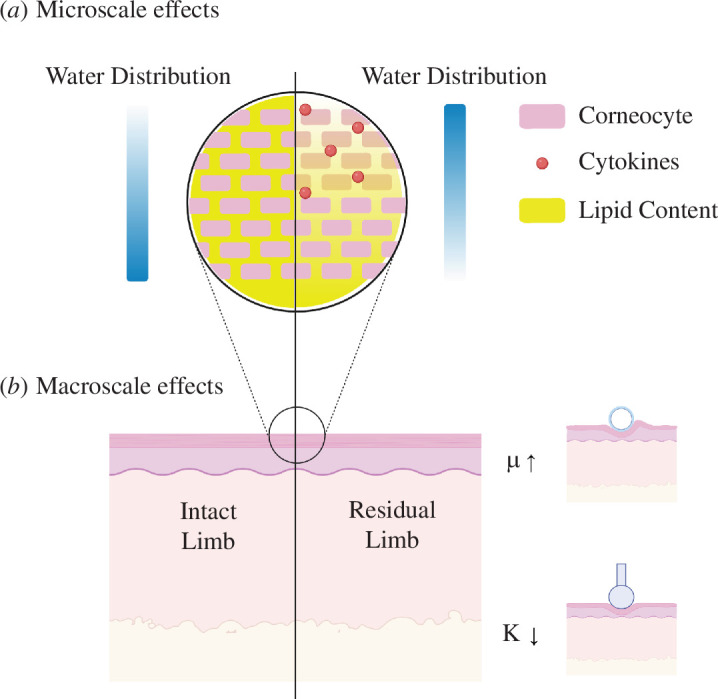
Summary of main results across the scales. (*a*) Microscale effects of prosthesis use on superficial skin’s barrier function and state of inflammation as measured by confocal Raman spectroscopy and cytokine analysis. (*b*) Macroscale effects of prosthesis use on bulk limb tissue mechanical properties as measured by an *in vivo* indenter and tribometer. The diagrams in (*a*) and (*b*) were created using BioRender.

This body of work shows that substantial adaptation of the skin does occur within an amputee’s limb (figure 5). These adaptations, however, do not suggest a higher resistance of the tissue to mechanical loading, as seen in our plantar heel measures. For lower limb amputees the rehabilitation journey is most hampered by socket fit and discomfort [59] whereby prosthesis use is limited by high skin injury rates [60], an issue that can continue throughout an amputee’s lifetime. Skin tolerance to mechanical load is known to vary by anatomical location [7], but current treatment plans are based on the belief that skin plasticity is such that residuum skin can be trained or conditioned to become load bearing like plantar skin. Here, a multi-scale approach has been taken to interrogate how skin at the residuum interface has changed from non-load-bearing skin, using the intact limb as a reference. This study highlights that amputee skin architecture has an interplay of factors across scales that lead to a higher susceptibility to injury. Reductions in residuum indentation mechanics and increased friction are thought to be driven by changes in skin barrier function, with water distribution, lipid content and NMF playing a pivotal role in reducing skin stiffness and increasing skin friction. As a result of softer mechanics and higher friction, higher internal strains lead to increases in inflammatory cytokines and a more wound-like phenotype. These findings are contrary to the current amputee skin adaptation theory highlighting that alternative therapies to ‘conditioning’ are required to reduce skin injury in the limb difference community.

## Data Availability

Data can be found at [61].
